# Cellular changes at the glia-neuro-vascular interface in definite idiopathic normal pressure hydrocephalus

**DOI:** 10.3389/fncel.2022.981399

**Published:** 2022-09-02

**Authors:** Per Kristian Eide

**Affiliations:** ^1^Department of Neurosurgery, Oslo University Hospital—Rikshospitalet, Oslo, Norway; ^2^Faculty of Medicine, Institute of Clinical Medicine, University of Oslo, Oslo, Norway

**Keywords:** idiopathic normal pressure hydrocephalus, aquaporin-4, pathophysiology, intracranial pressure, astrocytes, capillaries

## Abstract

Idiopathic normal pressure hydrocephalus (iNPH) is a subtype of dementia with overlap toward Alzheimer's disease. Both diseases show deposition of the toxic metabolites amyloid-β and tau in brain. A unique feature with iNPH is that a subset of patients may improve clinically following cerebrospinal fluid (CSF) diversion (shunt) surgery. The patients responding clinically to shunting are denoted *Definite* iNPH, otherwise iNPH is diagnosed as *Possible* iNPH or *Probable* iNPH, high-lightening that the clinical phenotype and underlying pathophysiology remain debated. Given the role of CSF disturbance in iNPH, the water channel aquaporin-4 (AQP4) has been suggested a crucial role in iNPH. Altered expression of AQP4 at the astrocytic endfeet facing the capillaries could affect glymphatic function, i.e., the perivascular transport of fluids and solutes, including soluble amyloid-β and tau. This present study asked how altered perivascular expression of AQP4 in subjects with definite iNPH is accompanied with cellular changes at the glia-neuro-vascular interface. For this purpose, information was retrieved from a database established by the author, including prospectively collected management data, physiological data and information from brain biopsy specimens examined with light and electron microscopy. Individuals with definite iNPH were included together with control subjects who matched the definite iNPH cohort closest in gender and age. Patients with definite iNPH presented with abnormally elevated pulsatile intracranial pressure measured overnight. Cortical brain biopsies showed reduced expression of AQP4 at astrocytic endfeet both perivascular and toward neuropil. This was accompanied with reduced expression of the anchor molecule dystrophin (Dp71) at astrocytic perivascular endfeet, evidence of altered cellular metabolic activity in astrocytic endfoot processes (reduced number of normal and increased number of pathological mitochondria), and evidence of reactive changes in astrocytes (astrogliosis). Moreover, the definite iNPH subjects demonstrated in cerebral cortex changes in capillaries (reduced thickness of the basement membrane between astrocytic endfeet and endothelial cells and pericytes, and evidence of impaired blood-brain-barrier integrity). Abnormal changes in neurons were indicated by reduced post-synaptic density length, and reduced number of normal mitochondria in pre-synaptic terminals. In summary, definite iNPH is characterized by profound cellular changes at the glia-neurovascular interface, which probably reflect the underlying pathophysiology.

## Introduction

Idiopathic normal pressure hydrocephalus (iNPH) is a subtype of dementia, with close histopathological overlap toward Alzheimer's disease (AD) regarding deposition of amyloid-β and tau in brain (Leinonen et al., 2010). The iNPH disease was even proposed to serve as a model disease of AD (Libard and Alafuzoff, 2019). Emerging evidence indicates that iNPH may be more prevalent than previously considered; two independent groups concluded that iNPH may occur in more than 5% of the population above 80 years (Jaraj et al., 2014; Andersson et al., 2019). The iNPH disease is a severe brain disease that is accompanied with increased 5-year mortality (Jaraj et al., 2017). However, a major challenge is that the iNPH disease is not well-characterized, not least reflected by the limitations of the current diagnostic guidelines (Relkin et al., 2005; Nakajima et al., 2021). For example, patients may fulfill the current diagnostic criteria without responding to the only available treatment, namely cerebrospinal fluid (CSF) diversion (shunt) surgery. Hence, the prediction of shunt response remains a challenge and a matter of debate, with the best predictive tests being invasive, costly and accompanied with risk (Thavarajasingam et al., 2021, 2022). Currently, the patients improving following shunt surgery may be considered the “real” iNPH patients, and who are denoted *definite iNPH* according to the Japanese guidelines (Nakajima et al., 2021). Understanding the underlying disease mechanisms in this subgroup of patients seems particularly important for deciphering iNPH pathophysiology, and may as well shed light on AD disease pathophysiology. Why are both diseases characterized by deposition of amyloid-β and tau?

**Figure 1:**
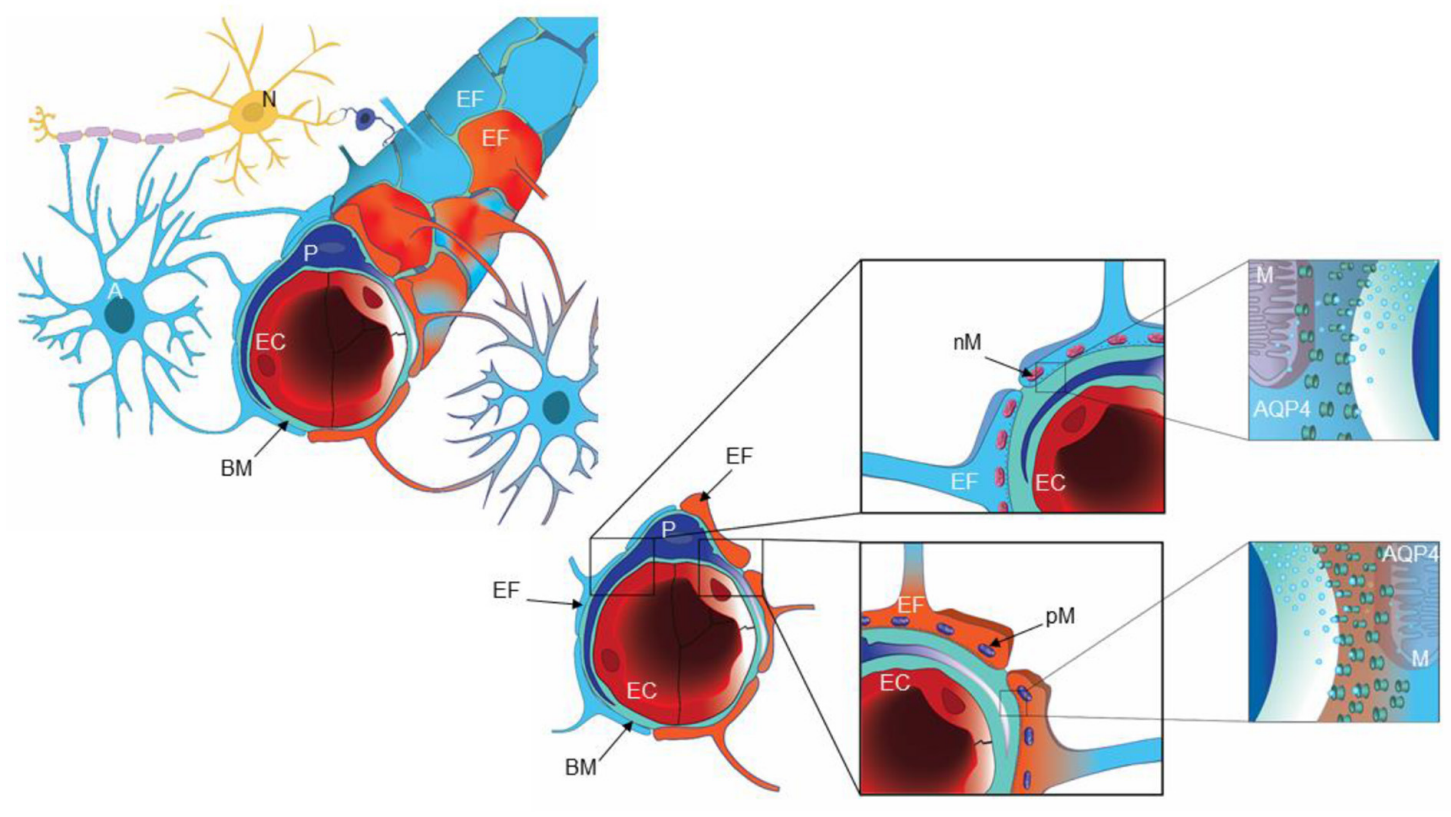
A cartoon illustrating the glia-neuro-vascular interface. The capillary (left) consists of endothelial cells (EC), pericytes (P) and the basement membrane (BM), which is a loose matrix between endothelial cells (EC) and pericytes (P). The endfeet (EF) of astrocytes (A) creates a donut shaped structure around the basement membrane (BM), separated by inter-endfeet-gaps between the endfoot (EF) processes. Normal endfeet (EF) are indicated in blue to the left while pathological endfeet (EF) in red color to the right. Astrocytic endfeet of iNPH patients presented a higher number of pathological mitochondria (PM) while REF subjects showed a higher number of normal mitochondria (NM). To the right is cartooned the water channel AQP4. Illustration: Ine Eriksen, University of Oslo.

Our group has previously provided evidence that pathology at the glia-neuro-vascular interface may be an essential component of iNPH pathophysiology (Eidsvaag et al., 2017b; Eide and Hansson, 2018; Hasan-Olive et al., 2019a,b). Within the adult human brain, the total length of capillaries has been estimated to about 600–700 km, covering a total surface area of 20 m^2^ (Winkler et al., 2014; Sweeney et al., 2016). Brain capillaries are organized in neurovascular units, also denoted the glia-neuro-vascular interface (Figure 1). This unit comprises a capillary built of endothelial cells with tight junctions and pericytes, a basement membrane lining the abluminal aspects of the endothelial cells and pericytes, and the perivascular astrocytic endfoot processes that form a donut shaped structure surrounding the basement membrane (Fernandez-Klett et al., 2010; Hall et al., 2014; Winkler et al., 2014; Sweeney et al., 2016). The pericytes enable dilatation and constriction of brain capillaries independent of arterioles (Fernandez-Klett et al., 2010; Iadecola, 2013; Hall et al., 2014; Mishra et al., 2016; Sweeney et al., 2016; Kisler et al., 2017). Moreover, the neurovascular unit (or coupling) is under neuronal control, providing for regulation of cerebral blood flow, the latter being impaired in iNPH (Ziegelitz et al., 2014, 2016).

Possible limitations with our previous studies on glia-neuro-vascular changes in iNPH are age differences between study and control groups and inclusion of consecutive iNPH patients of probably and possible types, given the diagnostic challenges of iNPH disease (Relkin et al., 2005; Nakajima et al., 2021). Furthermore, an interesting question not fully addressed in our previous studies, is how different alterations at the glia-neuro-vascular interface associate. This present study questioned how altered expression of the water channel aquaporin-4 (AQP4) at astrocytic perivascular endfeet in patients with definite iNPH is accompanied with cellular changes at the glia-neuro-vascular interface. AQP4 is the predominant water channel in the brain. Today, 13 isoforms of the water channel aquaporin have been identified; they serve as bidirectional facilitators of passive transport of water, small neutral solutes and certain ions across biological membranes, driven by osmotic or hydrostatic gradient for water transport and concentration gradient for solutes (Wagner et al., 2022). The isoforms present within the brain are AQP1 (choroid plexus) in addition to AQP4 (astrocyte processes, ependymal cells); both are mainly selective for water (Wagner et al., 2022). AQP4 is highly expressed at perivascular astrocytic endfeet facing the basement membrane of the capillaries, and plays a crucial role for brain water homeostasis (Nagelhus and Ottersen, 2013), and not least for glymphatic function (Iliff et al., 2012; Salman et al., 2021). Loss of AQP4 was accompanied with impaired glymphatic transport (Iliff et al., 2012).

In the present study, to further explore the role of AQP4 in iNPH, information was retrieved from a database (Neurovascular-Cerebrospinal fluid Quality registry, reg. no 2011/6692) established by the author since 2010, including clinical management data, physiological data, and information obtained as part of research, such as brain biopsy specimens. The present study included individuals in the registry categorized as definite iNPH, in whom brain biopsy data about AQP4 were available. In addition, individuals who matched the definite iNPH cohort closest in age and gender were included as reference (REF) subjects.

## Materials and methods

### Database, approvals, study design, and patient inclusion

The Neurovascular-Cerebrospinal fluid quality control database at the Department of neurosurgery, Oslo university hospital—Rikshospitalet, since 2010 included patients with neurovascular and CSF disturbances (Approval no. 2011/6692; Institutional Data Protection Official at Oslo university hospital). For iNPH patients, the registry stores prospectively collected clinical management information (preoperative symptoms and outcome results), imaging findings, over-night intracranial pressure (ICP) scores, as well as results of analyses of cortical brain biopsies. The morphological information about alterations at the glia-neuro-vascular interface is retrieved from cortical brain biopsies obtained as part of a research project approved by The Regional Committee for Medical and Health Research Ethics of Health Region South-East, Norway (Approvals no. REK 2009/2060, 2012/1157, and 2011/2306) and by Oslo University Hospital (Approvals no. 10/6806 and 2011/19311). The study is performed according to the ethical standards laid down in the 1964 Declaration of Helsinki and its later amendments. Patients are included after oral and written informed consent.

This present study asked how altered expression of the water channel AQP4 at perivascular astrocytic endfeet and neuropil in patients with definite iNPH is accompanied with cellular changes at the glia-neuro-vascular interface. For this purpose, information was retrieved from the database from the two following patient cohorts, included according to the following criteria:

- *Definite iNPH patients*. Inclusion criteria were: (1) The patients fulfilled the diagnostic criteria of definite iNPH according to the Japanese guidelines (Nakajima et al., 2021). (2) Information about AQP4 expression in cortical brain biopsies was available in the database. (3) Information about ICP scores was available in the database. Exclusion criteria were: (1) iNPH patients managed conservatively or categorized as shunt non-responders, i.e., not being definite iNPH. (2) Age > 75 years.
- *Reference (REF) subjects*. (1) Patients in the database in whom apparently healthy brain tissue was removed as part of otherwise planned brain surgery, i.e., neurosurgery for epilepsy, brain tumor or cerebral aneurysm (without prior bleeds). Notably, brain tissue resection was required as part of the necessary surgery, and the brain tissue specimens include apparently normal brain tissue not affected by the primary disease process. (2) AQP4 expression in the cortical brain biopsies had been determined. (3) Patients were matched as close as possible with definite iNPH subjects according to age and gender.

The patient material was established prior to analysis, to avoid selection bias. All examinations of ultrastructural changes in brain biopsies have been performed blinded to the diagnosis or other patient identifiable information.

### Variables retrieved from database

#### Demographic, clinical, and ICP data

Demographic information included age, gender, co-morbidity body (occurrence of arterial hypertension and/or diabetes mellitus), and preoperative symptoms.

**Figure 2:**
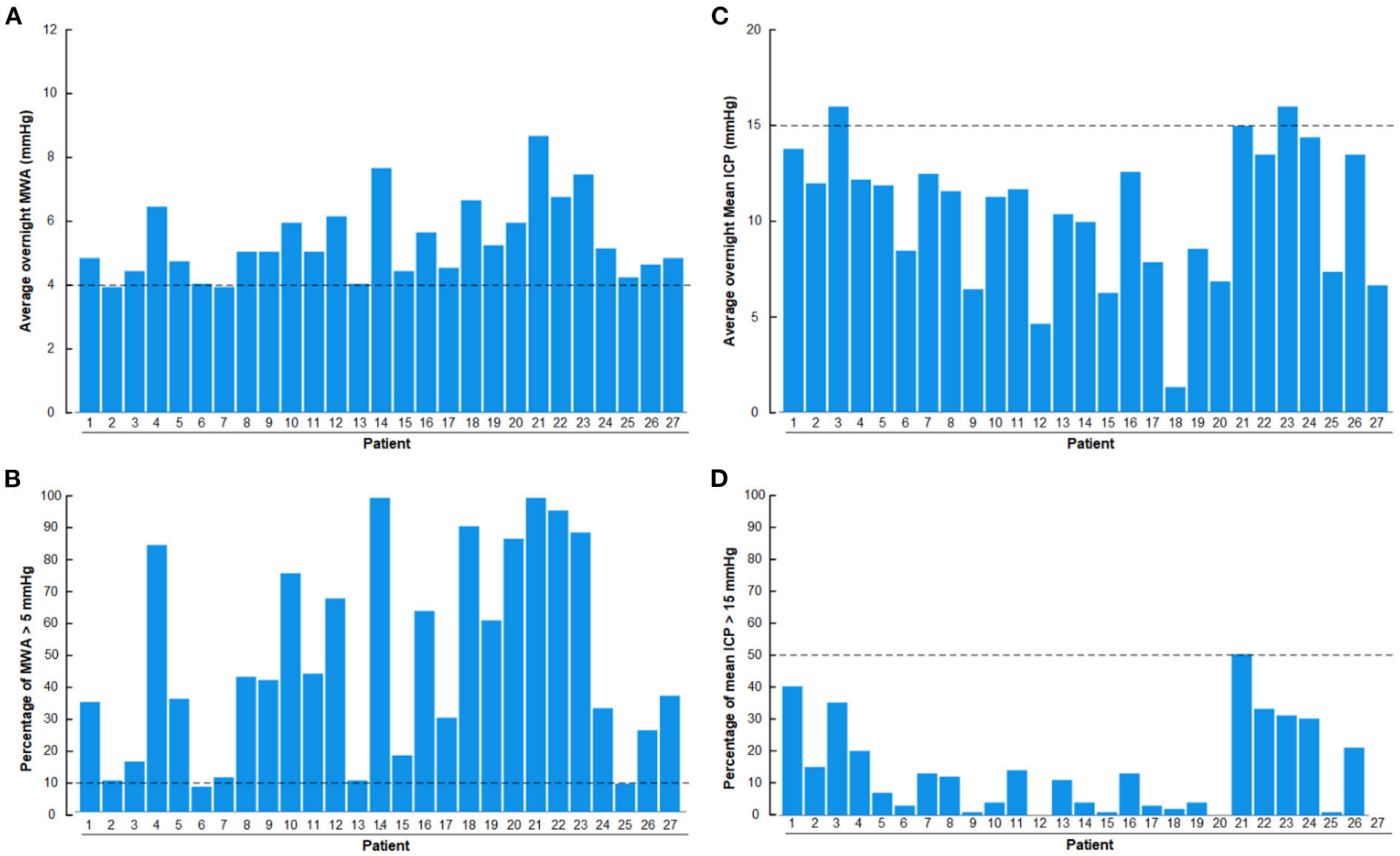
Overnight recordings of pulsatile ICP (mean wave amplitude; MWA) and static ICP (mean ICP) in the 27 patients with definite iNPH included in the study. **(A)** Overnight average of MWA. **(B)** Overnight percentage of MWA > 5 mmHg. **(C)** Overnight average of mean ICP. **(D)** Overnight percentage of mean ICP > 15 mmHg. The horizontal dashed lines show the upper normal thresholds as utilized by the first author: **(A)** Overnight MWA >4 mmHg. **(B)** Overnight 10% of MWA >5 mmHg. **(C)** Overnight Mean ICP > 15 mmHg. **(D)** Overnight 50% of Mean ICP >15 mmHg. The pulsatile ICP (MWA) was abnormal in the presently included definite iNPH patients.

Results of overnight ICP monitoring extracted from the database include overnight average of pulsatile ICP (mean ICP wave amplitude; MWA), percentage of MWA >5 mmHg, average of static ICP (mean ICP) and percentage of mean ICP >15 mmHg (Figure 2). The ICP had been measured using a ICP sensor (Codman MicroSensor^TM^, Johnson & Johnson, Raynham, MA, USA) introduced 1–2 cm into the frontal cortex parenchyma, as previously described (Eide and Sorteberg, 2010, 2016). We have previously defined abnormal pulsatile ICP during over-night monitoring as average MWA >4.0 mmHg and/or MWA >5.0 mmHg in >10% of recording time (Eide and Sorteberg, 2010).

#### Brain biopsy sampling and immunohistochemistry examinations

The surgical procedure of obtaining cortical brain biopsies in iNPH patients has been described previously (Eide and Hansson, 2018), and is only shortly commented on. A disposable Nashold Biopsy Needle (Integra Radionics, Burlington, MA, USA) was used to obtain a biopsy immediately below the cortical surface, providing for atraumatic and standardized biopsy sections. Biopsies from REF subjects were removed microsurgically from cerebral cortex. After tissue sampling, the brain biopsies were prepared for both light microscopy (LM) and transmission electron microscopy (TEM).

##### Light microscopy (LM)

The handling, processing and analysis of brain tissue for LM were done as previously described (Eide and Hansson, 2018). For LM, tissue specimens comprising the superficial three cortical layers (Layers 1–3) were fixed in buffered 4% paraformaldehyde for 2 days in the cold, dehydrated and embedded in paraffin, and tissue blocks were sectioned at a thickness of 6 μm. Following deparaffinization and rehydration, the sections were immersed in aqueous hydrogen peroxide for about 10 min to quench endogenous peroxidase activity. Antigen retrieval was obtained by immersion in proteinase K working solution for 10 min at pH 8.0; the subsequent blocking was obtained by normal horse serum before incubation over-night with the primary antibody at 4°C. After rinsing, a biotinylated antibody was applied over night at 4°C (Vectastain Universal Elite Kit; Vector Labs. Inc., Burlingame, CA, USA), followed by an avidin-biotin complex. Visualization was obtained with a 3,3′-diaminobezidine peroxidase substrate kit (Vector). Sections were counterstained with hematoxylin, and examined by LM after mounting and being cover-glassed.

Astrocytes were identified by their expression of aquaporin-4 (AQP4) and anti-gliofibrillary acidic protein (GFAP), nerve cell damage by the antibody neurofilament heavy (NF-H), and extravasated fibrin(ogen) expressed by an antibody revealing both fibrinogen and fibrin (polyclonal, 1:400, Dako, A0080), indicative of blood-brain-barrier (BBB) dysfunction. We used these primary antibodies: anti-AQP4 (polyclonal produced in rabbit against a recombinant protein tag, 1:1,000, Sigma); anti-dystrophin Dp71 (polyclonal produced in rabbit against a recombinant protein, 1:300, Abcam ab 15277); anti-gliofibrillary acidic protein (GFAP; 1:3,000, mouse monoclonal clone GA5, Sigma); anti-neurofilament heavy (NF-H, monoclonal against phosphorylated and non-phosphorylated neurofilaments, clone N52,1:500, Sigma); and anti-fibrin(ogen) (polyclonal, 1:400, A0080, Dako A/S, Glostrup, Denmark). In parallel, we processed reference sections, controlling the specificity of the immunohistochemical reaction by omission of the primary antibody.

The AQP4 expressions at astrocytic endfeet perivascular and toward neuropil were examined. Likewise, the expression of the anchoring molecule dystrophin (Dp71) was assessed. Semi-quantification of AQP4 and Dp71 expression was performed using densitometry analysis, providing an estimate of transmitted light, expressed as arbitrary units. Notably, higher arbitrary units is indicative of increased transmitted light, providing evidence for reduced AQP4 expression. Furthermore, astrocytic glial cells were examined for the presence of astrogliosis, astrocyte hypertrophy and loss of astrocyte domains (Wilhelmsson et al., 2006; Oberheim et al., 2012). The densitometry analysis and morphometry to determine percentage area of either GFAP immunoreactivity (3 areas per biopsy specimen, 273 × 410 μm) was done utilizing a Nikon Eclipse Ni microscope, a DS-Ri2 camera and NIS element B.V4.3 program (Nikon, Tokyo, Japan) and a Leica QWin Pro system (Leica Biosystems GmbH, Wetzlar, Germany). The semi-quantification and morphometry procedures were done by a person who was blinded to the diagnosis of the patients. Abnormal changes in neurons were assessed by the expression of neurofilament-H (NF-H), which is indicative of axonal damage. This is characteristic for damaged neurons while lack of NF-H immunoreactivity is typical for normal neurons. Leakage of the BBB was examined by determining area of fibrin(ogen) immunoreactivity in neuropil.

##### Transmission electron microscopy (TEM)

The TEM examinations has been described before (Eidsvaag et al., 2018; Eide et al., 2021), and are shortly commented on. The tissue specimens constituting at least the three deeper layers of the cerebral cortex (Layers 4–6) were immersion fixed in 0.1M phosphate buffer with 4 % paraformaldehyde and 0.25% glutaraldehyde. It was kept in a fridge (4°C) overnight, and thereafter transferred to the same fixative diluted 1/10 in phosphate buffer, and stored in the solution until further processing. Small blocks from the biopsies were cut, freeze substitution and infiltration in Lowicryl HM20 resin (Polysciences Inc., Warrington, PA, USA, Cat15924). The sections were counterstained with uranyl acetate during the cryosubstitution steps prior to the Lowicryl embedding. Using a Reichert ultramicrotome (Wien, Austria), sections of 80 nm were cut, and mounted on nickel grids. A FEI Tecnai^TM^ 12 transmission electron microscope (FEI Company, Hillsboro, OR, USA) was applied for TEM recording; images were acquired with analySIS image analysis software (Soft Imaging Systems GmbH, Münster, Germany). Cortical capillaries were defined as a vascular structures having an inner diameter <8 μm and a lumen bordered by a thin layer of endothelial cells (1–2 per circumference). Abluminally, the capillary was delimited by a basement membrane (BM) completely enclosing pericytes (cell body and processes) (Liwnicz et al., 1990; Farkas et al., 2000; Mathiisen et al., 2010; Alberts et al., 2014; Winkler et al., 2014; Sweeney et al., 2016). The astrocytic perivascular endfeet were defined by their position along the abluminal border of the BM. The structure of the astrocyte processes was assessed from the same brain biopsies utilizing both light and electron microscopic immunohistochemistry. Furthermore, for assessment of BM thickness, the BM membrane between astrocytic endfeet and endothelial cells (BM_E_) and the one between endfeet and pericytes (BM_P_) were measured separately around each capillary at an interval of 1–2 μm, and only at sites where the morphological quality was sufficient to obtain reliable measures. Average values of BM_E_ and BM_P_ were obtained for each capillary, requiring minimum six measures to be included.

For immunogold cytochemistry and determining density of AQP4 at astrocytic endfeet, the sections were incubated as follows: (1) 50 mM glycine in Tris buffer (5 mM) with 0.01% Triton X-100 and 50 mM NaCl (TBST; 10 min). (2) 2% Human serum albumin (HSA) in TBST (10 min). (3) Primary antibody (anti-AQP4 from Sigma-Aldrich; 25 μg/mL) diluted in the solution used in the preceding step (overnight). (4) Same solution as in step 2 (10 min × 2). (5) Gold-conjugated IgG (GAR15 nm, Abcam, Cambridge, UK), diluted 1:20 in TBST containing 2% HSA and polyethylene glycol (0.5 mg/mL, 1 h). Sections were then counterstained with 1% uranyl acetate and 0.3% lead citrate. AQP4 expression was analyzed as linear density of AQP4 toward endothelium, as previously described (Hasan-Olive et al., 2019c). Postsynaptic densities (PSDs) were identified in dendritic spines and measured by their length of electron dense appearance, as previously described (Eide et al., 2021). The post-synaptic density is a measure of synaptic strength. With regard to mitochondria, they were categorized as previously described (Hasan-Olive et al., 2019b): Normal mitochondria were dark and electron dense, showing regular shape with intact matrix and cristae. Pathological mitochondria were light and less electron dense, irregular in shape and with a swollen appearance and less intact matrix and cristae.

### Statistical analysis

Statistical analyses were performed using SPSS software version 27 (IBM Corporation, Armonk, NY); differences between Definite iNPH and REF groups were done with independent samples *t*-tests for continuous data, and with Pearson Chi-Square test for categorical data. Statistical significance was accepted at the 0.05 level.

## Results

### Patient material

The patient cohorts fulfilling the criteria included 27 subjects with definite iNPH and eight REF subjects (Table 1). The average age differed by one decade between the two cohorts (66.2 ± 5.3 vs. 55.6 ± 12.0), even though definite iNPH / REF individuals closest in age were included. As expected, the NPH scores differed between the patient groups (Table 1).

**Table 1:** Information about the study groups.

	**Definite iNPH**	**REF**	**Significance**
**Number**	27	8	
**Gender (F/M)**	11/16	4/4	ns
**Age mean at inclusion (yrs.)**	66.2 ± 5.3	55.6 ± 12.0	*P* = 0.001
**Co-morbidity**			
Arterial hypertension, *n* (%)	15 (55.6%)	4 (50%)	ns
Diabetes mellitus, *n* (%)	8 (29.6%)	1 (12.5%)	ns
**Pre-operative symptoms**			
Duration of symptoms	2.6 ± 1.7	12.0 ± 13.7	*P* = 0.001
Total NPH score	10.1 ± 1.6	14.6 ± 0.5	*P* <0.001
Gait NPH sub-score	3.0 ± 0.6	5.0 ± 0	*P* <0.001
Incontinence NPH sub-score	3.6 ± 0.8	5.0 ± 0	*P* <0.001
Cognitive NPH sub-score	3.5 ± 0.6	4.6 ± 0.5	*P* <0.001

F, Female; M, Male. Continuous data are presented as mean ± standard deviation, and categorical data as numbers (with percentages in parenthesis). Significant differences between continuous variables were determined by independent samples *t*-test, and differences between categorical data were determined by Pearson chi square test. Ns, non-significant.

With regard to the overnight ICP scores, there was abnormal pulsatile ICP in 27/27 (100%) of definite iNPH subjects, according to this authors previously defined abnormal thresholds (Eide and Sorteberg, 2010), i.e., overnight MWA scores >4 mmHg (Figure 2A) or overnight percentage of MWA > 5 mmHg in >10% of recording time (Figure 2B). The author has not previously defined normal thresholds of abnormal mean ICP, but tentatively define abnormal thresholds as over-night mean ICP > 15 mmHg (Figure 2C) or overnight mean ICP > 15 mmHg in >50% of recording time (Figure 2D). According to these thresholds, abnormal mean ICP was seen in 2/27 (7%) iNPH patients. They were, however, not categorized as pressure hydrocephalus since increased mean ICP was not accompanied with markedly increased MWA.

### Comparisons of ultrastructural variables at glia-neuro-vascular interface

The technical procedure of brain biopsy was not accompanied with adverse consequences in any of the included patients. In the 27 definite iNPH patients, the brain biopsies were obtained from right frontal cerebral cortex. The eight REF subjects had undergone surgery for epilepsy (*n* = 4), resection of a malignant brain tumor (*n* = 1), or clipping of a cerebral aneurysm without prior bleed (*n* = 3). In these subjects, the brain biopsies were obtained from cerebral cortex frontal lobe in four and temporal lobe in four subjects.

**Figure 3:**
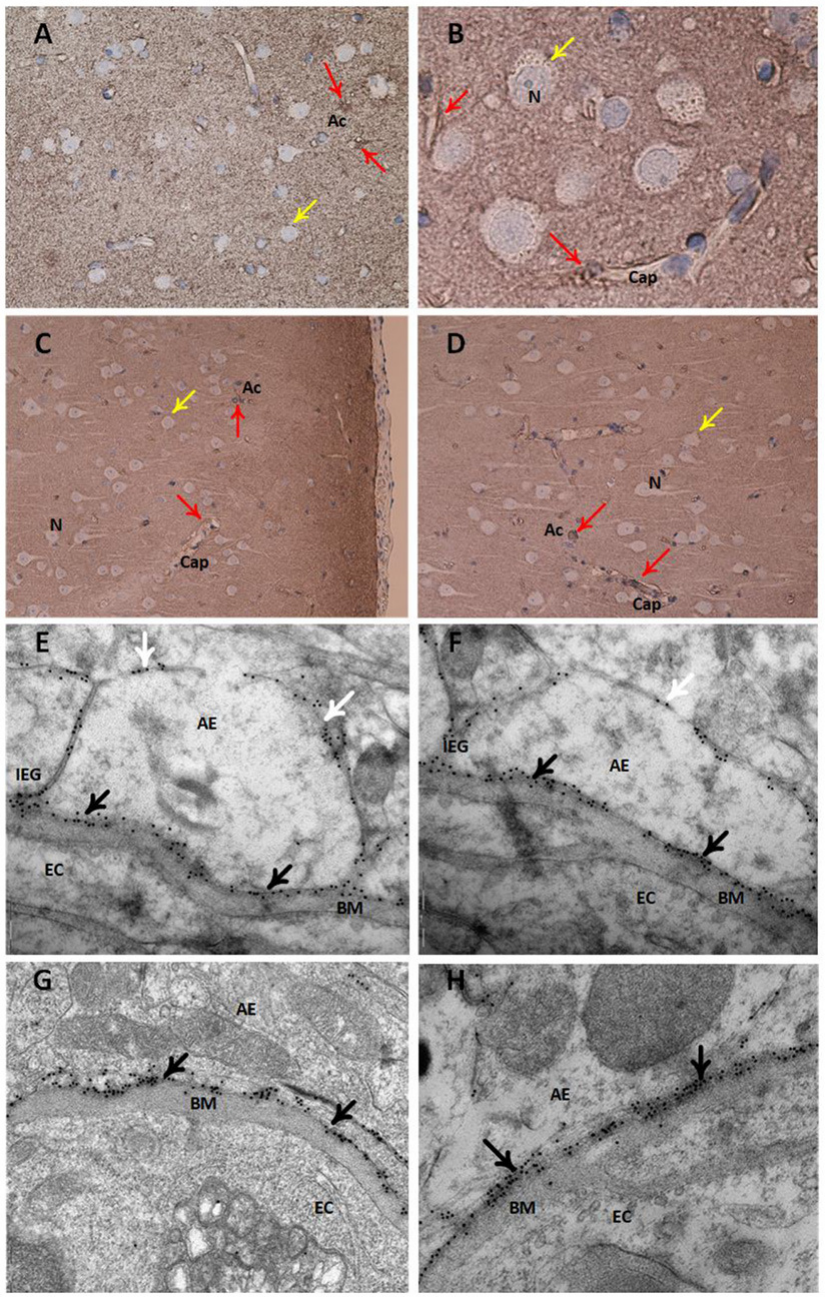
Expression of the water channel aquaporin 4 (AQP4) in astrocytes. Panels **(A–D)** present light microscopic visualization AQP4 immunoreactivity (IR). **(A,B)** In cortex layers 2–3 of definite iNPH subjects, neurons (yellow arrow) lack AQP4-IR while cell bodies and processes of astrocytes (red arrow) show AQP4-IR. Capillaries (Cap) are distinctly outlined by AQP4 IR in the astrocyte endfeet. **(C,D)** In a REF subject, the perivascular end feet lining capillaries are more intensely reactive than the neuropil. Panels **(E–H)** present transmission electron microscopy (TEM) of AQP4 immunogold labeling. **(E,F)** The AQP4 immunogold labeling (black arrow) at astrocytic endfeet (AE) is toward the basement membrane (BM) facing the endothelial cells (EC) in definite iNPH patients. There is a polarized AQP4 distribution with more AQP4 labeling toward basement membrane (black arrow) than toward neuropil (white arrow). The inter-endfeet gaps (IEG) between astrocytic endfeet (AE) processes are indicated. **(G,H)** The AQP4 immunogold labeling toward the basement membrane is shown in a REF subject. AE, Astrocytic endfoot; As, Astrocytes; BM, Basement membrane; Cap, Capillaries; EC, Endothelial cell; IEG, Inter-endfeet gaps; N, Neurons.

Semi-quantitative assessment of LM specimens demonstrated reduced AQP4 immunoreactivity (i.e., increased arbitrary unit values) at perivascular borders and toward neuropil in iNPH vs. REF subjects (Table 2). Similarly, TEM immunogold labeling showed reduced density of AQP4 at astrocytic membranes facing the capillary basement membrane and toward neuropil (Table 2). There was a significant negative correlation between immunogold AQP4 expression at perivascular astrocytic endfeet and AQP4 immunoreactivity toward blood vessels (Pearson correlation −0.54, *P* = 0.02; data not shown), i.e., reduced AQP4 expression shown by LM was correlated with reduced linear density of AQP4 at astrocytic endfoot membranes shown by TEM. Images of LM and TEM findings are shown in Figure 3.

**Table 2:** Expression of AQP4 at perivascular astrocytic endfeet and membranes facing neuropil in definite iNPH and REF patients.

	**Definite iNPH**	**REF**	**Significance**
**LIGHT MICROSCOPY**			
**Perivascular astrocytic endfeet**			
**AQP4 at membranes facing endothelial cells**			
LM—Arbitrary units	102.8 ± 13.0	67.3 ± 5.4	*P* <0.001
Areas with vessels lacking perivascular AQP4 IR (yes/no)	24/0	1/6	*P* <0.001
**AQP4 at membranes facing neuropil**			
LM—Arbitrary units	137.6 ± 19.4	117.6 ± 6.5	*P* = 0.013
**ELECTRON MICROSCOPY**			
**Endfoot membrane facing endothelial cell**			
No of observations	32.5 ± 13.5	26.1 ± 5.5	ns
Length (μm) per observation	2.3 ± 0.3	2.3 ± 0.4	ns
AQP4—Linear density (Particles/μm)	14.7 ± 3.1	18.5 ± 2.2	*P* = 0.009
**Parenchymal membrane (i.e. facing neuropil)**			
No of observations	16.6 ± 10.6	8.3 ± 8.9	ns
Length (μm) per observation	2.2 ± 0.2	2.2 ± 0.3	ns
AQP4—Linear density (Particles/μm)	6.0 ± 2.1	8.5 ± 2.1	*P* = 0.021
AQP4 Polarization index	2.8 ± 1.4	2.3 ± 0.7	ns

Continuous data presented as mean ± standard deviation, and categorical data as numbers. Significant differences between groups were determined by independent samples *t*-test for continuous data and by Pearson Chi-Square test for categorical data (ns, non-significant).

**Figure 4:**
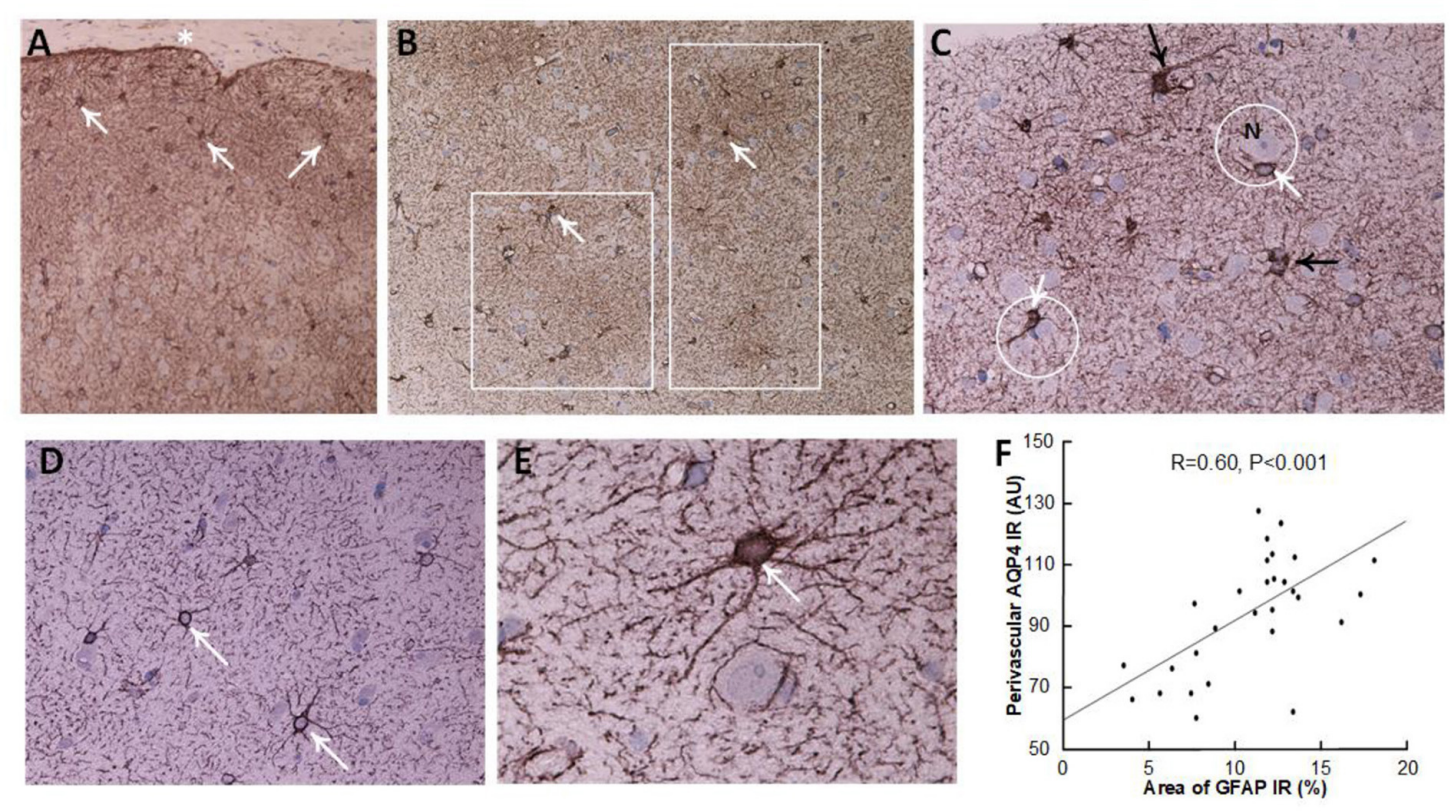
Astrocytes visualized by GFAP IR in brain cortex biopsies. **(A–C)** A definite iNPH patient. **(A)** Low magnification of a brain biopsy section shows a high prevalence of astrocytes (white arrows) in the subpial molecular layer (also denoted Chaslin's layer). Pia indicated by an asterisk. **(B)** Patches of astrogliosis are shown at a higher magnification, indicated by white boxes. White arrows indicate astrocytes. **(C)** Hypertrophic astrocytes are enclosing unstained neurons (N), encircled by a white line. Processes from adjacent astrocytes enter their neighbors' domains and present with increased branching (black arrows). **(D,E)** In a REF subject, normal astrocytes (white arrows) present with normal domains. **(F)** There was a highly significant positive correlation between GFAP immunoreactivity (IR) and AQP4 arbitrary units (AU), indicating that with increasing astrogliosis (stronger GFAP expression) there was reduced expression of AQP4 at perivascular astrocytes (higher arbitrary units). The plot includes the fit line and Pearson correlation coefficient with significance level.

Definite iNPH is characterized by astrogliosis, here shown as increased area of GFAP immunoreactivity, astrocyte hypertrophy and loss of astrocyte domains (Table 3 and Figure 4). Increasing area of GFAP IR, indicative of increased astrogliosis, is accompanied with reduced expression of perivascular AQP4 (i.e., higher arbitrary units; Pearson correlation 0.60, *P* < 0.001; Figure 4F).

**Table 3:** Assessment of astrocytes from brain biopsies of definite iNPH and REF patients.

	**Definite iNPH**	**REF**	**Significance**
Area of GFAP immunoreactivity (%)	12.0 ± 3.0	7.6 ± 2.9	*P* = 0.002
Astrocyte hyperthrophy (yes/no)	24/0	3/4	*P* <0.001
Distinct astrocyte domains (yes/no)	1/23	5/2	*P* <0.001

GFAP, Glial fibrillary acidic protein. Continuous data presented as mean ± standard deviation, and categorical data as numbers. Significant differences between groups were determined by independent samples *t*-test for continuous data and by Pearson Chi-Square test for categorical data.

**Figure 5:**
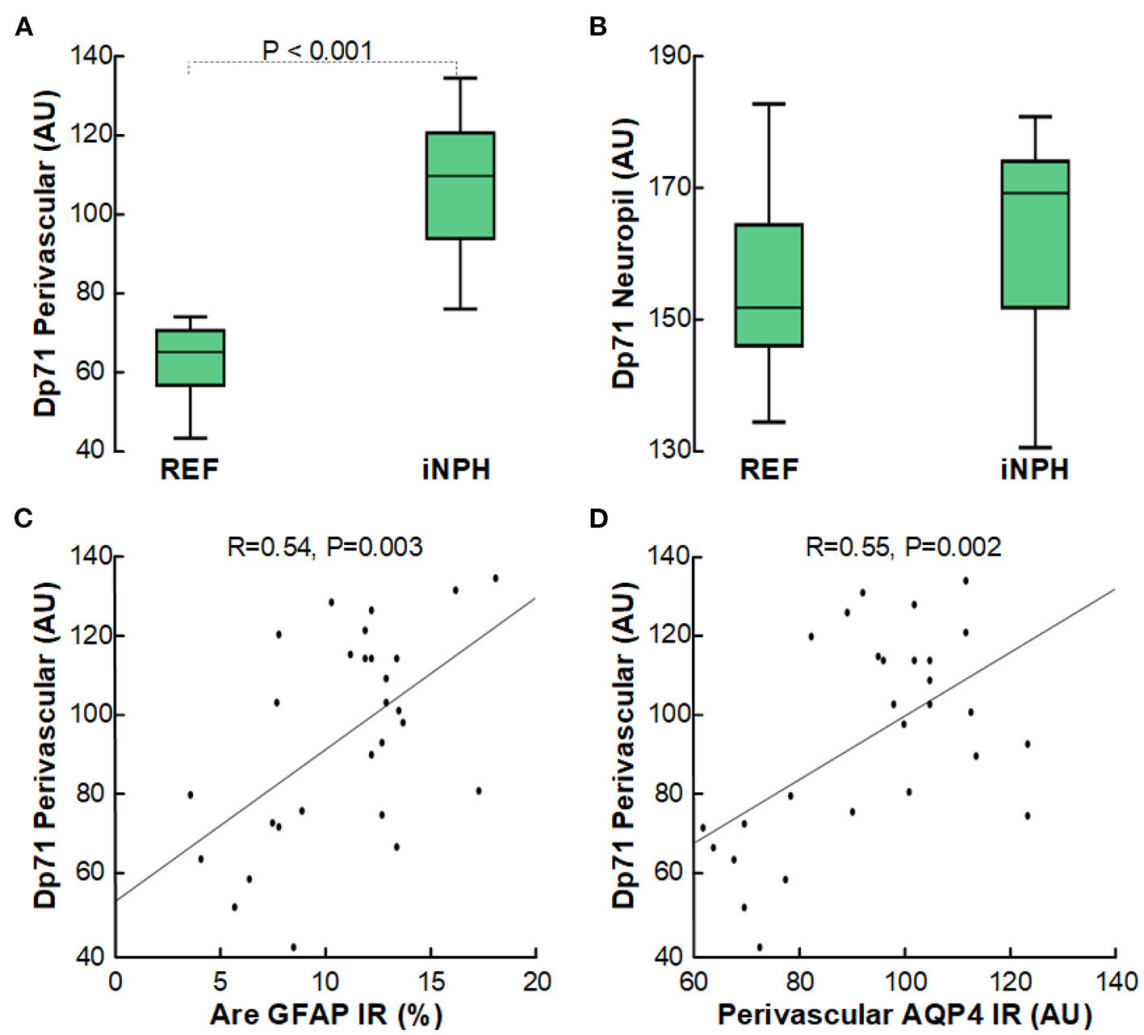
Reduced expression of perivascular Dp71-IR in cerebral cortex of definite iNPH subjects. **(A)** In definite iNPH patients, the Dp71 arbitrary units (AU) were significantly higher perivascular, indicative of lower Dp71 expression. **(B)** The definite iNPH and REF groups did not differ concerning Dp71 arbitrary units in neuropil. **(C)** There was a highly significant positive correlation between DP71 AU and area of GFAP IR, indicating that with increasing astrogliosis there was reduced perivascular Dp71 expression. **(D)** There was a highly significant positive correlation between perivascular Dp71 and perivascular AQP4 arbitrary units (AU), indicating that with reduced perivascular Dp71 expression, also perivascular AQP4 expression was reduced. The plots show the fit line and Pearson correlation coefficient with significance level.

Furthermore, it is of note that the loss of perivascular AQP4 was accompanied with reduced expression of perivascular Dp71 (shown as increased arbitrary units of perivascular Dp71), though not for Dp71 toward neuropil (Figures 5A,B). As for AQP4, increasing degree of astrogliosis was accompanied with reduced perivascular Dp71 (Figure 5C), and there was significant correlation between loss of perivascular AQP4 and Dp71 (i.e., reduced expression of AQP4 was accompanied with reduced expression of Dp71; Figure 5D).

**Figure 6:**
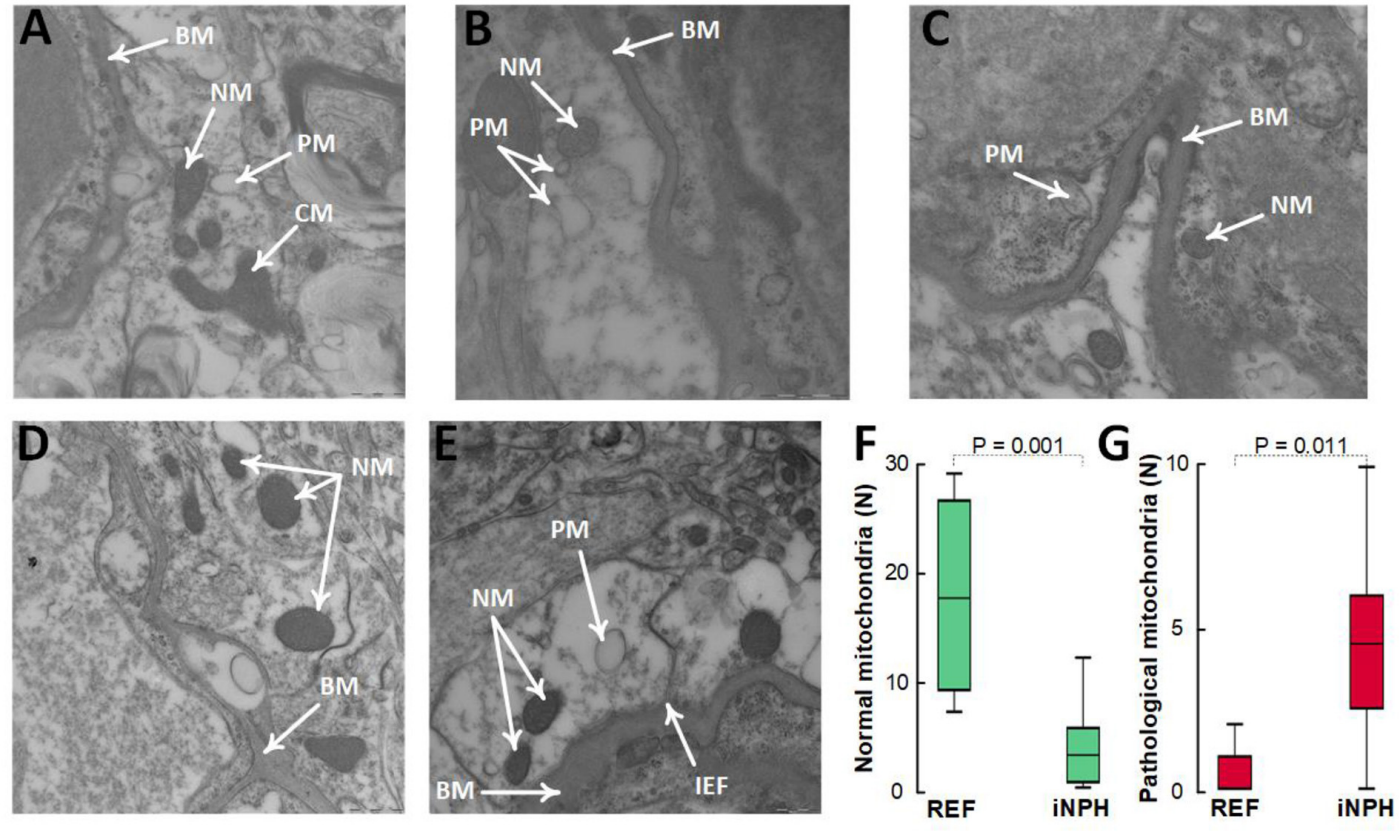
Evidence for reduced proportion of normal mitochondria and higher proportion of pathological mitochondria in perivascular astrocytic endfeet of definite iNPH patients. **(A–C)** In astrocytic endfeet facing the basement membrane (BM) of definite iNPH patients, there were higher proportion of pathological mitochondria (PM) and lower proportion of normal mitochondria (NM). **(D,E)** REF subjects showed higher proportion of normal mitochondria (NM) in astrocytic endfeet facing the basement membrane (BM). Differences between definite iNPH and REF subjects concerning number of normal mitochondria **(F)** and pathological mitochondria **(G)** were determine by independent samples *t*-test.

In these definite iNPH patients, there was evidence of impaired cellular metabolism in perivascular astrocytic endfeet facing the capillary basement membrane, shown as reduced number of normal mitochondria and higher number of pathological mitochondria in the astrocytic endfeet (Figures 6A–G).

**Figure 7:**
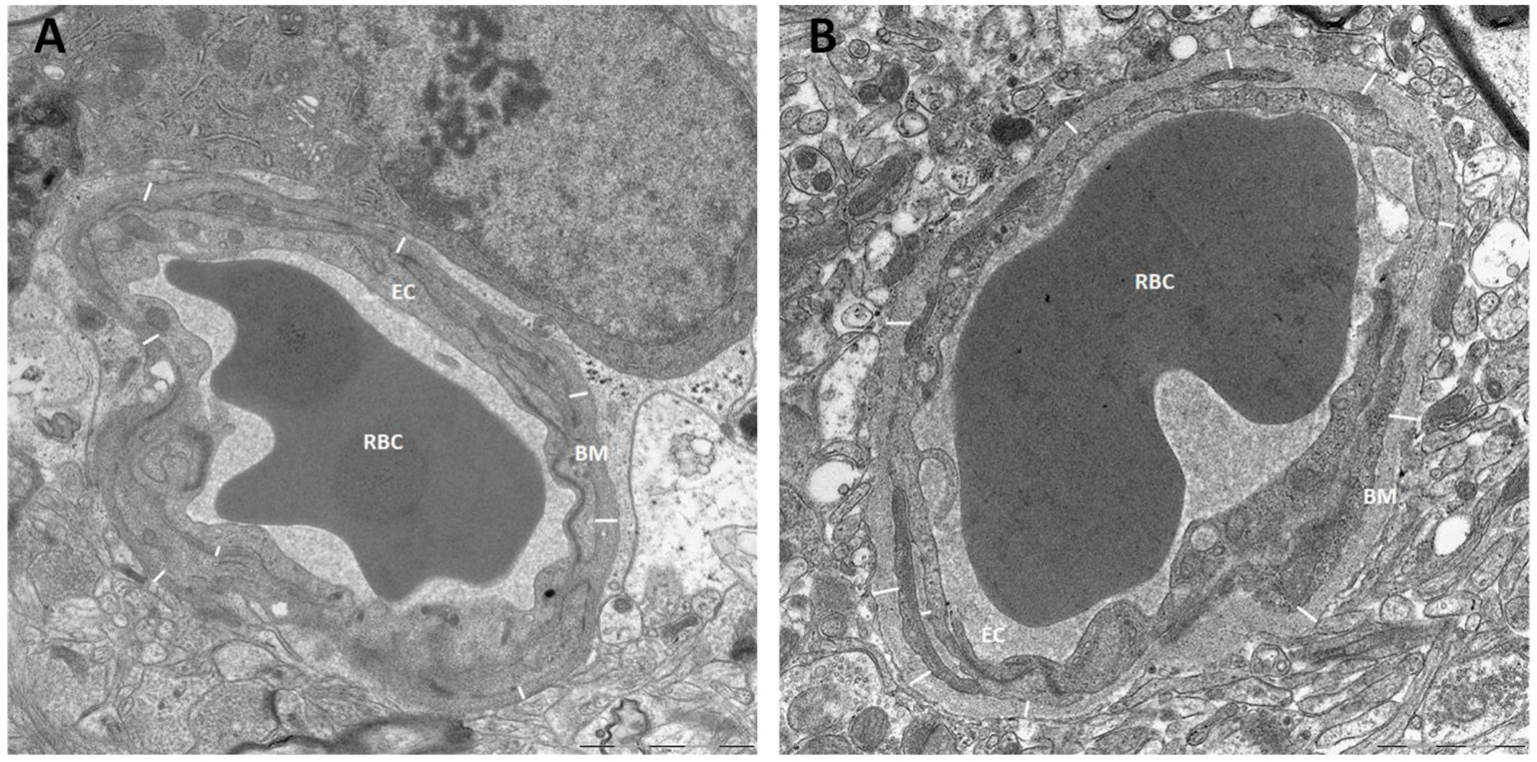
Basement membrane thickness was reduced in definite iNPH patients. **(A)** The thickness of basement membrane (BM) toward endothelial cells (EC) was measured at multiple sites for each capillary, here from a definite iNPH subject. **(B)** The basement membrane thickness shown for a REF subject. BM, Basement membrane; RBC, Red blood cell; EC, Endothelial cell.

The thickness of the capillary basement membrane was measured at multiple sites around the capillary circumference (Figure 7). Both the thickness of the basement membrane between endfeet and endothelial cells (BM_E_) and the thickness of the basement membrane between astrocytes and pericytes (BM_P_) were reduced in definite iNPH patients (Table 4). Hence, in definite iNPH patients with loss of perivascular AQP4, the basement membrane in reduced in thickness.

**Table 4:** Thickness of basement membrane in definite iNPH and REF patients.

	**Definite iNPH**	**REF**	**Significance**
**BM**_**E**_ **(Astrocytic endfeet—Endothelial cells)**			
Capillaries (*n*)	69	31	
Thickness (nm)	222.1 ± 58.5	328.5 ± 122.4	P = 0.022
**BM**_**P**_ **(Astrocytic endfeet—Pericytes)**			
Capillaries (*n*)	65	26	
Thickness (nm)	197.8 ± 39.9	294.8 ± 125.1	P = 0.023

Continuous data presented as mean ± standard deviation. Significant differences between groups were determined by independent samples *t*-test.

**Figure 8:**
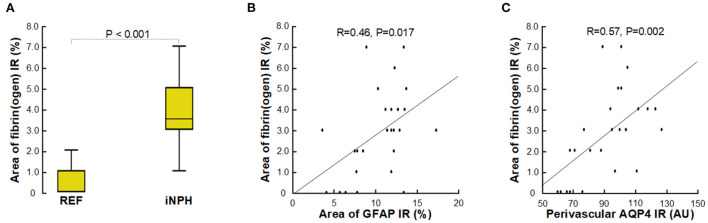
Assessment of impaired BBB integrity with leakage of fibrin(ogen). **(A)** The fibrin(ogen) immunoreactivity (IR) in neuropil is indicative of impaired BBB integrity, and was increased in definite iNPH patients as compared with REF subjects. **(B)** There was a highly significant positive correlation between area of fibrin(ogen) IR and area of GFAP IR and **(C)** between fibrin(ogen) IR and perivascular AQP4, indicating that with increased BBB leakage there was increased astrogliosis, and that with increased BBB leakage perivascular AQP4 expression was reduced. The plots show the fit line and Pearson correlation coefficient with significance level.

Another observation indicative of capillary changes in definite iNPH was increased expression of the blood proteins fibrin(ogen) in neuropil, which reflects extravasation of fibrin(ogen) to neuropil due to impaired blood-brain-barrier integrity (Figure 8A). Of note, extravasation of fibrin(ogen) to neuropil is not normal in healthy children or adult and indicates impaired integrity of the BBB (Petersen et al., 2018; Sweeney et al., 2018b). Fibrin(ogen) is pro-inflammatory and there was a significant correlation between increased area of fibrin(ogen) in neuropil and increased area of GFAP, indicative of astrogliosis (Figure 8B). Moreover, with increasing degree of fibrin(ogen) extravasation, there was reduced expression of perivascular AQP4 (Figure 8C).

**Figure 9:**
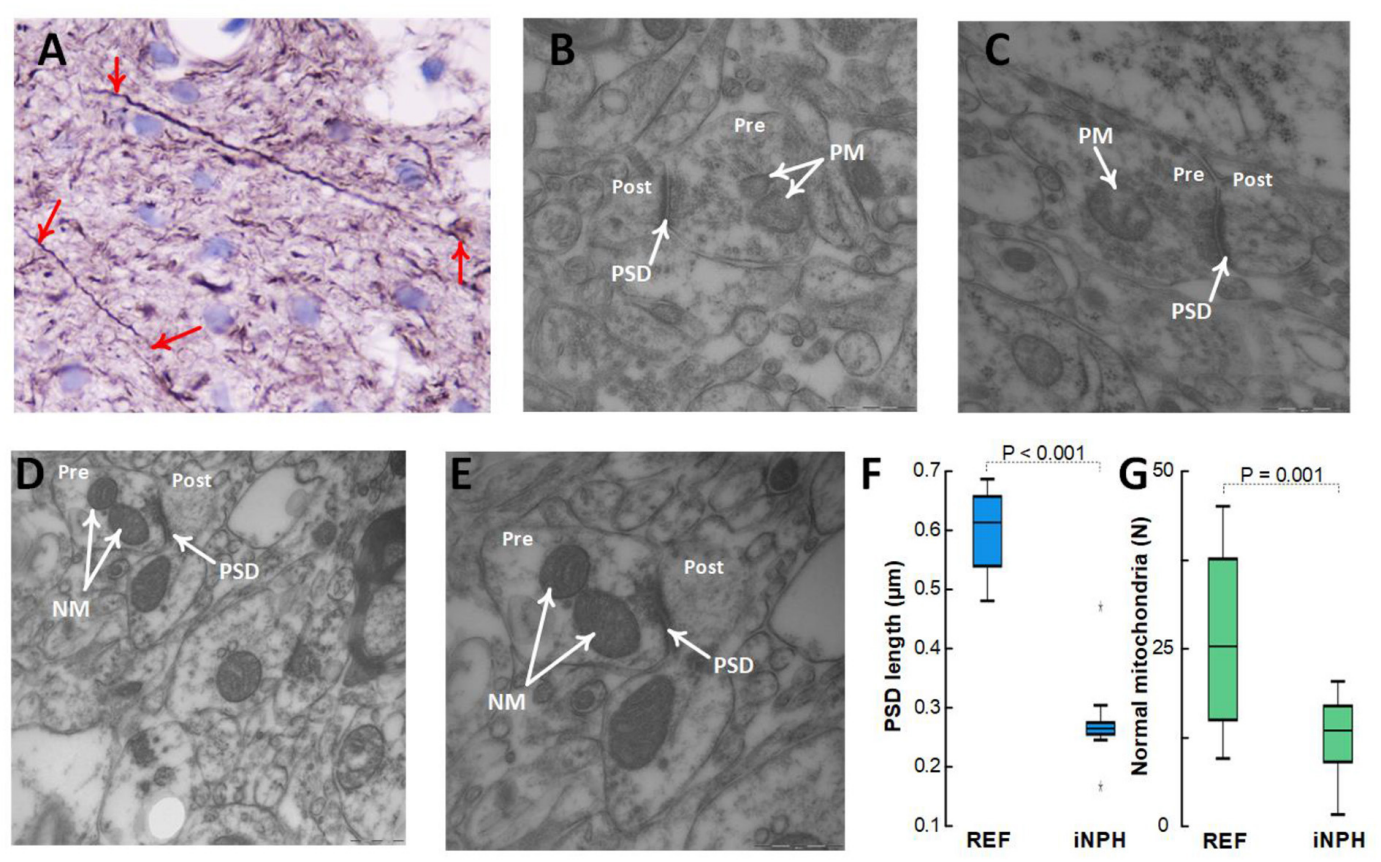
Assessment of neuronal markers in the cerebral cortex. **(A–C)** Definite iNPH patient. **(A)** There is neurofilament-H immunoreactivity expression in damaged or dying neurons (red arrows) of a definite iNPH patient, indicative of neuronal damage in definite iNPH. Notably, the longitudinally orientated nerve processes show an irregular outline and focal swellings (beads) and retraction balls that are indicative of disturbed axonal transport and degeneration. In this material, positive neurofilament-H IR was seen in 24/24 (100%) iNPH patients and in 1/7 (14%) REF subjects (*P* < 0.001, Pearson Chi square test). **(B,C)** The postsynaptic density (PSD) length (arrow) in definite iNPH was reduced, indicative of reduced synaptic strength and pathology. Pathological mitochondria (PM) in presynaptic terminals (Pre) are shown for definite iNPH subjects. **(D,E)** REF subject. Normal mitochondria (NM) in presynaptic terminals (Pre) are shown, also demonstrating the postsynaptic (Post) nerve terminals. **(F)** The PSD length was reduced in definite iNPH patients as compared with REF subjects. **(G)** The number of normal mitochondria in presynaptic terminals was reduced in definite iNPH patients. Statistical differences are indicated and were determined by independent samples *t*-test.

Patients with definite iNPH is further characterized by changes in neurons. Thus, there were positive neurofilament-H immunoreactivity in 24/24 (100%) of definite iNPH patients while in 1/7 (14%) of REF subjects (*P* < 0.001, Pearson Chi square test; Figure 9A). The expression of neurofilament-H immunoreactivity is seen in damaged or dying neurons, indicative of neuronal damage. TEM observations further showed shortened length of post-synaptic density in definite iNPH as well as reduced number of normal mitochondria in presynaptic terminals of definite iNPH (Figures 9B–G).

## Discussion

The present observations disclose reduced expression of the water channel AQP4 at both perivascular astrocytic endfeet and toward neuropil in cerebral cortex of subjects with definite iNPH compared to controls. This observation was accompanied with evidence of altered cellular metabolic activity in astrocytic endfeet shown as altered number of normal and pathological mitochondria in endfoot processes toward capillaries. The reduced expression of AQP4 was accompanied with reduced expression of the anchoring protein dystrophin (Dp71) at perivascular astrocytic endfeet. The degree of reduced expression was correlated with degree of astrogliosis in cerebral cortex. Furthermore, the definite iNPH subjects demonstrated in cerebral cortex reduced thickness of the basement membrane between astrocytic endfeet and endothelial cells and between endfeet and pericytes, as well as evidence of impaired BBB integrity shown as increased area of fibrin(ogen) IR in neuropil. Finally, definite iNPH presents with evidence of altered neuronal function, demonstrated by altered post-synaptic density, altered number of normal mitochondria in pre-synaptic terminals. Accordingly, loss of perivascular AQP4 in definite iNPH is accompanied with marked cellular changes at the glia-neuro-vascular interface.

This study exclusively included patients with definite iNPH according to the Japanese guidelines (Nakajima et al., 2021). Our previous studies addressing ultrastructure at the glia-neuro-vascular interface in iNPH included iNPH patients treated conservatively and surgically (both responders and non-responders), leaving a question about the situation in “true” iNPH, i.e., definite iNPH. Another limitation of our previous reports on this topic was the age difference between study and control groups approaching three decades (Eidsvaag et al., 2017b; Eide and Hansson, 2018, 2020; Hasan-Olive et al., 2019a). It could be questioned whether the observed findings were a consequence of aging. The control patients included in the database generally is of younger age than the iNPH patients, which limit a perfect age matching between study and controls. However, in this present study, the average age difference was one decade (66.2 ± 5.3 vs. 55.6 ± 12.0 years). It seems hard to explain the present results by such minor difference in age, though some effect of age cannot be excluded. The present results relate to definite iNPH and not necessarily iNPH in general.

Since 2002, we have measured the pulsatile ICP in iNPH patients overnight using conventional ICP sensors and dedicated software to quantify the continuous pulsatile ICP as mean ICP wave amplitude (MWA), and the ordinary static ICP as mean ICP. A consistent observation is that the MWA is abnormally elevated in the iNPH patients responding to shunt surgery. The present subjects with definite iNPH presented with abnormally elevated MWA scores. From early experience, we determined upper normal thresholds for MWA, referring to an average of the MWA overnight > 4 mmHg and with MWA > 5 mmHg in >10% of recording time (Eide and Sorteberg, 2010). Using these thresholds 9/10 patients experienced clinical improvement following shunting, while 1/10 with below-threshold MWA had shunt response (Eide and Sorteberg, 2010). For this, the MWA, not mean ICP, has been used as a supplementary test to predict shunt response in iNPH in our department. A recent systematic review and meta-analysis comparing invasive tests for predicting shunt response in iNPH reported the most accurate predictive tests in the following order: Pulsatile ICP measurements (here referred to as MWA estimations) > extended lumbar drainage > infusion tests > tap tests (Thavarajasingam et al., 2021). The abnormally elevated MWA becomes reduced with CSF diversion, either by extended lumbar drainage or by shunt surgery (Eide and Sorteberg, 2008; Eide and Stanisic, 2010). The increased pulsatile ICP in iNPH has been attributed to impaired intracranial compliance (i.e., reduced intracranial pressure volume reserve capacity; Eide, 2016), which may as well be intimately related to the events occurring at the glia-neuro-vascular interface. To which degree the observed cellular changes are downstream to the increased pulsatile ICP needs to be further explored.

The phenomenon of cerebral paravascular solute and fluid transport gained renewed interest since 2012 after introduction of the so-called “glymphatic system”—a pseudo-lymphatic system within the brain (Iliff et al., 2012). This system refers to a peri- (or para-)vascular pathway for transport of CSF along vessels at the artery side, mixing with interstitial fluid, enabling clearance of solutes along veins to the CSF. It is believed that the system is primarily active during sleep (Xie et al., 2013), and becoming impaired with increasing age (Kress et al., 2014). Furthermore, it has been suggested that the glymphatic system represents a final common pathway for neurodegenerative diseases, for example impaired perivascular efflux of amyloid-β and tau in Alzheimer's disease and impaired perivascular efflux of α-synuclein in Parkinson's disease (Nedergaard and Goldman, 2020). While the glymphatic system was first described in rodents, recent experimental evidence provides evidence that it is even more developed in the gyrencephalic brain of pigs (Bèchet et al., 2021). In patients with iNPH, we found evidence of impaired glymphatic function utilizing an intrathecal CSF tracer visualized by magnetic resonance imaging (MRI) (Ringstad et al., 2018; Eide and Ringstad, 2019), though MRI lacks the resolution (1 mm) to exactly define the route for tracer movement.

The glymphatic system is considered to heavily depend on intact AQP4 water channels at the astrocytic endfeet facing capillaries (Iliff et al., 2012). In this regard, it is important to bear in mind that glymphatic transport refers to movement of solutes outside cells, which is not synonymous with the transport of water (H_2_O) that occurs both para-cellular and across cell membranes. Normal water homeostasis is, however, a requirement for proper glymphatic function. AQP4 is the predominant water channel in the brain (Nagelhus and Ottersen, 2013); about 50% of the area of the astrocytic endfeet toward the basement membrane is covered by AQP4 (Nagelhus and Ottersen, 2013). In the plasma membrane, AQP4 tetramers aggregate into large supra-molecular square arrays, known as orthogonal arrays of particles (OAPs) (Nagelhus and Ottersen, 2013).

Events such as trauma and stroke may alter AQP4 expression, causing abnormal water distribution and disturbed ion balance (Nagelhus and Ottersen, 2013). While AQP4 is highly polarized to astrocytic perivascular endfoot processes in rodents (Nielsen et al., 1997), in human brain AQP4 expression is less polarized with a relatively high proportion of AQP4 in astrocyte membranes toward the neuropil (Eidsvaag et al., 2017a). The present observations showing loss of perivascular AQP4 in definite iNPH extend previous observations in different categories of iNPH patients (Eide and Hansson, 2018; Hasan-Olive et al., 2019a), providing evidence for more extensive reduction of AQP4 in astrocytic endfeet of definite iNPH, not only restricted to perivascular endfeet facing capillaries but also in endfeet membranes toward the neuropil (Hasan-Olive et al., 2019a). In addition, the present observations disclose significant differences between study and control groups differing about one decade in age, as compared with previous reports with about three decades in age difference between groups. The reduction of AQP4 expression in iNPH cannot solely be explained by higher age of this group. Another observation strengthening the validity of the results is the significant positive correlation between reduced perivascular AQP4 expression seen in LM and the quantitative measures of AQP4 immunogold labeling in TEM specimens. Therefore, the present data strengthen the evidence of loss of perivascular AQP4 in iNPH patients, indicating that it may be even more pronounced in definite iNPH who responds to CSF diversion (shunt) surgery.

Previously, various animal models have provided evidence that loss or mislocalization of AQP4 affect neurological disease development (Nagelhus and Ottersen, 2013; Papadopoulos and Verkman, 2013). In humans, loss of AQP4 at perivascular astrocytic endfeet was seen in several brain diseases, including mesial temporal lobe epilepsy (Eid et al., 2005), neuromyelitis optica (Misu et al., 2007), and ischemic stroke (Steiner et al., 2012; Stokum et al., 2015). In Alzheimer's disease, AQP4 expression was reduced or even lost around amyloid β deposits (Hoshi et al., 2012; Zeppenfeld et al., 2017). Experimentally, loss of AQP4 caused reduced clearance of amyloid-β from mouse brain (Iliff et al., 2012). Currently, the physiological role of the AQP4 water channel at perivascular astrocytic endfeet remains a topic of debate. Given the role of AQP4 in regulation of astrocyte volume, AQP4-mediate influx of water to the astrocytic endfeet processes may have an important role in the volume regulation of the endfeet processes. Hence, it was shown in cell culture of primary human cortical astrocytes that hypothermia caused astrocyte swelling accompanied with increased surface localization of AQP4 on the astrocytes, an effect that was blocked by a transient receptor potential vanilloid 4 calcium channel (TRPV4) antagonist (Salman et al., 2017). This is of interest given the previous experimental evidence that a TRPV4/AQP4 complex is essential for astrocytic cell volume regulation (Benfenati et al., 2011).

With regard to the elevated pulsatile ICP characterizing definite iNPH, perhaps the regulation of the volume and structure of the donut-shaped perivascular astrocytic endfeet have a cushioning effect of the pulsatile pressure changes created by the capillary pulsations? Even though not presently proven, dysfunctional astrocytic endfeet may be a less efficient pressure pulsation absorber, causing the elevated pulsatile ICP in definite iNPH. Accordingly, the dysfunctional astrocytic endfeet may represent a biological explanation of the impaired pulsation absorber mechanisms and abnormal pulsatile ICP previously reported in iNPH subjects (Park et al., 2012).

One may as well speculate that the abnormalities at the astrocytic endfeet interfere with the regulation of the size of the inter-endfeet gaps, which were previously estimated to about 20 nm (Mathiisen et al., 2010). The size of the inter-end-feet gaps probably determines the efflux of molecules from the perivascular basement membrane to the interstitial space. This could in turn affect the perivascular fluid transport capability. In iNPH, there is delayed efflux of a CSF tracer from the brain, presumably the perivascular compartment (Ringstad et al., 2018), as well as redistribution of CSF flow (Eide et al., 2020). Moreover, the increased intracranial pulsatility may in turn restrict the arterial pulsatility. As such, reduced arterial pulsatility is accompanied with reduced perivascular solute transport (Mestre et al., 2018).

The present observations further confirm that loss of perivascular AQP4 is accompanied with a wide range of other alterations at the glia-neuro-vascular interface. There was a significant correlation between loss of perivascular AQP4 and loss of perivascular Dp71, which is the major isoform of dystrophin in the brain. This protein connects the cell's cytoskeleton with the membrane and the extracellular matrix (Waite et al., 2012; Nagelhus and Ottersen, 2013), and is part of a dystrophin-associated protein complex (DAPC) in the membrane of the astrocytic perivascular endfeet that anchors the water channel AQP4 as well as ion channels (Nagelhus and Ottersen, 2013). Loss or mislocalization of proteins in the DAPC and AQP4 deteriorate water fluxes to/from the endfeet and impair cerebral metabolism (Nagelhus and Ottersen, 2013; Pavlin et al., 2017). Targeted deletion of a member of the DAPC removes a substantial proportion of perivascular AQP4 pool (Hoddevik et al., 2017). Another significant aspect is the emerging evidence that subcellular re-localization of AQP4 from intracellular vesicles to the plasma membrane is essential for AQP4 function, which occurs dynamically and independent of changes in AQP4 expression (Kitchen et al., 2020). Evidence has been given that the mechanisms behind sub-cellular re-localization of AQP4 represents a potential target for drugs, for example inhibitors of AQP4 function (Kitchen et al., 2020; Markou et al., 2022; Salman et al., 2022). To further explore the alterations of AQP4 function in neurological diseases such as iNPH, new tools such as human microvessel-on-a-chip platforms and 3D cultures may become useful (Wevers et al., 2018; Salman et al., 2020). In this regard, real-time assessment of changes in AQP4 may provide new insight as compared to the static information of microscopy images. Today, the exploration and discovery of potential new pharmaceuticals targets and therapies, e.g. to modify AQP4 function in iNPH disease, is aided by new methods such as efficient high-throughput screening (HTS) and computer-aided drug design (Aldewachi et al., 2021).

A most significant observation is that definite iNPH patients showing loss of perivascular AQP4 also presented with evidence of altered cellular metabolism at perivascular astrocytic endfeet. Hence, the number of normal mitochondria was reduced while the number of pathological mitochondria was increased in perivascular astrocytic endfeet of definite iNPH, supporting previous results from different iNPH categories separated about three decades in age from controls (Hasan-Olive et al., 2019b). Previous studies utilizing 3-dimensional (3D) TEM showed bundles of mitochondria in the astrocytic processes nearby perivascular endfoot membrane (Mathiisen et al., 2010). This further adds support to dysfunctional perivascular astrocytic endfeet in definite iNPH.

The endfeet processes and their molecular composition are as well affected by astrogliosis (Eid et al., 2005; Heuser et al., 2012). Presently, there was a significant correlation between the degree of loss of perivascular AQP4 and the degree of astrogliosis, semi-quantified by area of GFAP. The astrogliosis also was characterized by cell hypertrophy and loss of astrocyte domains. Astrogliosis refers to a non-specific alteration of astrocytes, occurring secondary to e.g., chemokines, cytokines, infections and inflammations, noxious agents, and trauma (Oberheim et al., 2012; Verkhratsky and Butt, 2013; Winters and Kleinschmidt-Demasters, 2015). The astrogliosis is accompanied with increased stiffness of the reactive glia cells, correlating with increased expression of cytoskeletal structures (Lu et al., 2011). These changes may in turn increase the stiffness of the brain and reduce its compliance. In definite iNPH, astrogliosis may have a profound impact on brain function, given that the glial cells, including the astrocytes, constitute about half of the total number of brain cells and half of the adult brain volume (Verkhratsky and Butt, 2013; Winters and Kleinschmidt-Demasters, 2015). It is now established that the astrocytes play a key role in brain metabolism (Howarth, 2014). In the present context, the perivascular astrocytic endfeet are crucial for vasomotion and water and fluid homeostasis (Amiry-Moghaddam and Ottersen, 2003; Mulligan and MacVicar, 2004; Boulay et al., 2017; Langer et al., 2017), and for maintenance of BBB function (Haddad-Tovolli et al., 2017).

Another intriguing observation in definite iNPH is that loss of perivascular AQP4 was accompanied with thinner basement membrane both toward endothelial cells and toward pericytes. Thickness of basement membrane in definite iNPH was around 200 nm (Table 4). In comparison, capillaries in the normal adult human retina, which is considered a part of the brain, had a basement membrane thickness of close to 300 nm (Bianchi et al., 2016). This finding of thinner basement membrane in definite iNPH might be surprising given that basement membrane thickness was found increased in conditions such as arterial hypertension, diabetes, and brain edema (Junker et al., 1985; Farkas et al., 2000; Farkas and Luiten, 2001; Onodera et al., 2012; Castejon, 2014; Bianchi et al., 2016; Sweeney et al., 2016), and also in dementia diseases (Claudio, 1996; Farkas et al., 2000). Furthermore, several studies show that human basement membrane thickness increases with aging (Farkas et al., 2000; Uspenskaia et al., 2004; Powner et al., 2011; Bianchi et al., 2016). On the other hand, no difference in basement membrane thickness was seen in a previous study comparing iNPH patients with REF subjects separated more in age and incorporating a higher proportion of epilepsy patients as controls (Eidsvaag et al., 2017b). Epilepsy is known to be associated with thickened basement membrane and accelerated brain aging (Liwnicz et al., 1990; Thom et al., 2011). The extracellular matrix of the basement membrane consists of molecules such as collagen, laminin, agrin, perlecan, and fibronectin (Farkas et al., 2000; Alberts et al., 2014; Thomsen et al., 2017), which interact with DAPC that serves as anchor to AQP4 and the potassium channel Kir4.1 (Nagelhus and Ottersen, 2013).

The thinning of the basement membrane in definite iNPH was accompanied with leakage of the blood glycoprotein fibrin(ogen), a large molecule with a molecular weight of 340 kDa, being a marker of BBB integrity, not demonstrable in the normal adult human brain parenchyma but in trace amounts in elderly (Alafuzoff et al., 1985, 1987; Paul et al., 2007; Sweeney et al., 2016, 2018a,b; Medcalf, 2017; Petersen et al., 2018). Fibrinogen, which is transformed to fibrin outside the blood vessels, is pro-inflammatory and promotes inflammation (Alafuzoff et al., 1985; Paul et al., 2007; Zlokovic, 2008; Sengillo et al., 2013; Sweeney et al., 2016; Liebner et al., 2018). In iNPH, arterial hypertension and diabetes mellitus are well-known vascular risk factors (Eide and Pripp, 2014). Previous studies have shown reduced cerebral blood flow (Ziegelitz et al., 2014, 2016) and low-grade ischemia (Eide and Stanisic, 2010; Calcagni et al., 2013) in iNPH, indicative of impaired cerebrovascular function in this disease.

The microvascular changes at the capillary level may heavily affect neuronal function. In humans, the median inter-capillary distance is about 50 μm and the distance between a capillary and a neuron is about 10 μm. In the brain, there is one capillary for each neuron. The present observations disclosed more prevalent neurofilament H expression and axonal changes (torpedoes and beaded axons) in definite iNPH, which is indicative of disturbed axonal transport and neuronal degeneration (Tang-Schomer et al., 2012; Verkhratsky and Butt, 2013). Moreover, definite iNPH demonstrated reduced post-synaptic density length as well as reduced number of normal mitochondria in presynaptic terminals. Together, these observations point at impaired neuronal function in definite iNPH, which is of interest given that cognitive impairment is part of diagnostic criteria of iNPH (Nakajima et al., 2021). It has previously been reported that mitochondrial trafficking and distribution is connected to synaptic activity (MacAskill and Kittler, 2010). Furthermore, a sufficient number of functional mitochondria is a requirement for maintenance of normal synaptic function due to the high-energy demand in the pre- and post-synaptic terminals (Hollenbeck, 2005; Yu and Yu, 2017). Thus, the present observations of reduced postsynaptic density length in iNPH is of interest, given that postsynaptic density is a measure of the strength of the synaptic activity. In this context, it is of note that oligomeric amyloid-β in close proximity to the postsynaptic area may shrink the postsynaptic density length, reduce synaptic plasticity and increase synaptic loss (Koffie et al., 2009).

### Limitations

Some limitations with the study should be noted. It might be considered a limitation that the study includes a rather low number of patients (27 vs. 8 patients). On the other hand, group differences were seen despite the rather low number of subjects. Another limitation is that cortical biopsy was obtained from the frontal lobe in all iNPH subjects while in the frontal (*n* = 4) and temporal (*n* = 4) lobes in REF subjects. Further studies are needed to address whether biopsy location would affect the results. Furthermore, given the small size of the brain biopsy (0.9 × 10 mm), it may be discussed to which extent the present observations reflect the situation within the entire brain. For that purpose, autopsy of whole brain specimens would be required. It may as well be criticized that reference subjects are not healthy controls, but patients undergoing neurosurgery for various reasons. This limitation may, however, be impossible to overcome since brain biopsy cannot be obtained from healthy controls for ethical reasons. On the other hand, the cortical biopsy was not taken from the brain area with disease, but from apparently healthy brain tissue that had to be removed as part of the necessary neurosurgery. Finally, even though the age difference between study and control groups was merely one decade in the present study, a role of aging for the present results may not be entirely excluded. It seems less likely, however, that the brain deteriorates extensively from average age 55–65 years. This age difference may hardly explain the presently reported group differences. Nevertheless, future studies should address the impact of aging on the observed results.

## Conclusions

The present data provide evidence that loss of the water channel AQP4 at membranes of astrocytic endfeet toward both capillaries and neuropil in definite iNPH is accompanied with extensive cellular changes at the glia-neuro-vascular interface. These alterations include loss of the anchoring protein Dp71, astrogliosis, cellular energy failure at astrocytic endfeet, capillary changes characterized by reduced basement membrane thickness and BBB leakage, as well as neuronal dysfunction. It is suggested that these changes play a pivotal role in the pathophysiology behind definite iNPH.

## Data availability statement

The raw data supporting the conclusions of this article will be made available by the authors, without undue reservation.

## Ethics statement

The studies involving human participants were reviewed and approved by the Regional Committee for Medical and Health Research Ethics of Health Region South-East, Norway. The patients/participants provided their written informed consent to participate in this study.

## Author contributions

PE: conceptualization and design, data analysis, writing—original draft, review and editing, supervision and administration, approval of the final manuscript, and correspondence and material requests.

## Funding

The work involving histopathological assessment of brain tissue specimens was supported by Grants from Health South-East, Norway (Grants 2012016 and 2016027).

## Conflict of interest

The author declares that the research was conducted in the absence of any commercial or financial relationships that could be construed as a potential conflict of interest.

## Publisher's note

All claims expressed in this article are solely those of the authors and do not necessarily represent those of their affiliated organizations, or those of the publisher, the editors and the reviewers. Any product that may be evaluated in this article, or claim that may be made by its manufacturer, is not guaranteed or endorsed by the publisher.

## Abbreviations

A: astrocyte
AD: Alzheimer's disease
AQP4: aquaporin-4
BBB: blood-brain-barrier
BM: basement membrane
CSF: cerebrospinal fluid
DAPC: dystrophin associated protein complex
Dp71: dystrophin
EC: endothelial cell
EF: endfoot
GFAP: glial fibrillary acidic protein
HTS: high-throughput screening
ICP: intracranial pressure
iNPH: idiopathic normal pressure hydrocephalus
LM: light microscopy
M: mitochondria
MRI: magnetic resonance imaging
MWA: mean ICP wave amplitude
N: neuron
NF-H: neurofilament-heavy
PSD: post-synaptic density
REF: reference
TEM, transmission electron microscopy, TRPV4: transient receptor potential vanilloid 4 calcium channel
VP: ventriculoperitoneal.
